# Digital health interventions for healthy ageing: a qualitative user evaluation and ethical assessment

**DOI:** 10.1186/s12877-021-02338-z

**Published:** 2021-07-02

**Authors:** Marcello Ienca, Christophe Schneble, Reto W. Kressig, Tenzin Wangmo

**Affiliations:** 1 Department of Health Sciences and Technology, ETH Zurich, Hottingerstrasse 10, HOA H17 Zürich, Switzerland; 2 Competence Centre for Rehabilitation Engineering and Science, ETH Zurich, Zürich, Switzerland; 3 Institute for Biomedical Ethics, University of Basel, Basel, Switzerland; 4 Department of Geriatric Medicine FELIX PLATTER, University of Basel, Basel, Switzerland

## Abstract

**Background:**

Digital health technologies are being increasingly developed with the aim of allowing older adults to maintain functional independence throughout the old age, a process known as *healthy ageing*. Such digital health technologies for healthy ageing are expected to mitigate the socio-economic effects of population ageing and improve the quality of life of older people. However, little is known regarding the views and needs of older people regarding these technologies.

**Aim:**

The aim of this study was to explore the views, needs and perceptions of community-dwelling older adults regarding the use of digital health technologies for healthy ageing.

**Method:**

Face-to-face, in-depth qualitative interviews were conducted with community-dwelling older adults (median age 79.6 years). The interview process involved both abstract reflections and practical demonstrations. The interviews were transcribed verbatim and analyzed according to inductive content analysis.

**Results:**

Three main themes and twelve sub-themes addressing our study aim resulted from the data obtained. The main themes revolved around favorable views and perceptions on technology-assisted living, usability evaluations and ethical considerations.

**Conclusions:**

Our study reveals a generally positive attitude towards digital health technologies as participants believed digital tools could positively contribute to improving their overall wellbeing, especially if designed in a patient-centered manner. Safety concerns and ethical issues related to privacy, empowerment and lack of human contact were also addressed by participants as key considerations.

**Supplementary Information:**

The online version contains supplementary material available at 10.1186/s12877-021-02338-z.

## Introduction

As a consequence of global population ageing, both the total size and the relative proportion of older people are steadily growing worldwide. In 2019, 703 million people in the world were aged 65 or older, that is approximately 9 % of the global population. By 2050 this proportion is projected to rise to 16 % with the total number reaching 1.5 billion [1]. Due to a parallel reduction in fertility and the improvements in survival, the global average life expectancy at birth (for both sexes) has reached 72.3 years, with peaks above 84 years in countries such as Italy (84.01), Singapore (84.07), Switzerland (84.25), Japan (85.03) and Hong Kong (85.29) [1].

These demographic trends are causing important shifts in the age structure of global societies. One of these shifts is captured by the change over time of the old-age dependency ratio (OADR), which is defined as the number of people aged 65 or older per hundred people aged between 20 and 64 (working age). In 2019, there were 16 older people per hundred working-age people globally. In Europe, the proportion of people aged 65 or older has already passed 30 per 100 people aged 20–65. By 2050, the OADR is expected to dramatically rise to 49 per 100 (as compared to 30 per 100 today) [1].

The upward shift in age distribution attested by the OADR is expected to fundamentally transform economic, social, cultural and political life throughout the world. One consequence of this transformation is increased healthcare expenditures as the probability of being sick and the associated costs of treatment are higher in the old age. Further, as the proportion of people in working age is shrinking in most countries, the sustainability of public finances (including healthcare services) will be jeopardized by, simultaneously, lower tax revenue and social security contributions, and higher social expenditures.

One approach towards mitigating these socio-economic effects of population ageing is creating the intrinsic, social and environmental conditions to allow older adults to maintain functional independence throughout the old age. *Healthy ageing* is the umbrella term typically used to refer this “process of developing and maintaining the functional ability that enables wellbeing in older age” (WHO 2019). Constitutive features of healthy ageing include maintaining mobility, meeting one’s own basic needs independently, learning and making decisions as well as building and maintaining relationships, and contributing to society.

In recent years, a variety of digital health solutions have been developed with the aim of supporting people in old age and promoting healthy ageing. Some of these digital tools are purposively designed to meet the needs and wishes of older people, and are thereby called ‘gerontechnologies’. Other tools are designed for the general population, but often embed features specifically designed for seniors. Gerontechnologies and other digital health solutions can assist older people in the completion of cognitive or physical tasks such as activities of daily living. This subset of digital health applications is often referred to as *intelligent assistive technology* (IAT) and it is of particular value in the assistance and support of older people with dementia or other age-related cognitive disability [2, 3]. Other digital health technologies do not provide any specific assistive function but can generate the necessary information to implement assistive functions. These include digital tools (software, hardware or both) for self-monitoring, activity tracking, or other applications aimed at measuring physiological, cognitive or fitness-related metrics such as heartbeat, calorie consumption, daily steps, reaction time at cognitive tests etc.

While the domain of digital health applications for healthy ageing is rapidly expanding in volume and variety, experts have observed several translational challenges that currently hinder the successful clinical translation and societal adoption of these technologies. These challenges include insufficient information-sharing and knowledge transfer among relevant stakeholders (e.g. between developers and clinicians), scarce clinical validation of new technologies and an insufficient consideration of user needs and perspectives in product design [2, 3]. The latter problem is well exemplified by the relative lack of user-centered design and assessment of IATs for older people [3] and confirmed by expert assessments by professional caregivers [4].

To this purpose, in this study, we conducted a qualitative user evaluation of four digital health systems for healthy ageing: a toy-shaped conversational robot, a smartphone application for care coordination, and two wrist-worn wearable devices. We incorporated this user evaluation in a broader user-centered qualitative assessment of digital health technologies for healthy ageing with special focus on assessing the interviewees’ perspectives on ethical considerations. This user evaluation and ethical assessment is necessary to complement previous ethics assessment studies involving proxy decision-makers (e.g. informal and formal caregivers) [4, 5] with first-hand information from older persons.

## Methods

This study is part of a larger research project entitled “Digitalizing Elderly Care in Switzerland: Opportunities and Challenges” funded by the Käthe Zingg-​Schwichtenberg Fund of the Swiss Academy of Medical Sciences (KZS20/17). In this study part, we conducted semi-structured qualitative interviews and usability evaluations with cognitively healthy older adults living in Switzerland. This qualitative explorative approach was used to identify predictors of adoption and to further understand what considerations should be incorporated into existing digital health solutions to improve effectiveness, safety, ethical alignment and user-friendliness among older users.

### Study sample

Since the study aim was to capture digitalization in the older population, the participants were all aged over 65 years and had to meet the following inclusion criteria denoted in Table 1. Participants were purposely recruited in-person at the Basel University Department of Geriatric Medicine “FELIX PLATTER”. They were given information about the study and if they returned the consent form, their contact details were forwarded to the research team. In total 20 interviews were conducted. As one participant decided to drop out after the interview, a total of 19 interviews were included in the data analysis. All participants included in the study were community-dwelling older adults (defined as individuals aged 65 or older who live independently in their own homes). Table 2 gives a demographic overview of the participants. Most participants had fairly good familiarity with and were regular users of basic personal computing technologies such as PCs, laptops and smartphones. Participants who reported not using a smartphone gave the following reasons: visual difficulties, cognitive difficulties, discouragement by others. Tables 3 and 4 provide an overview of participants’ use of personal digital technologies and their reported reasons for not using smartphone devices.

**Table 1:** Inclusion Criteria

Inclusion Criteria
Aged 65 and older
Living in Switzerland
Home-dwelling or living in institutional facilities
Speaking: Swiss German, German, French, Italian or English.
Mini-Mental State Examination (MMSE) score > 24

**Table 2:** Demographics

Demographic characteristics	N
Total Number of Participants	19
Gender (Female/Male)	9 (F) 10 (M)
Age (mean)	79.6 years
Children	15 (yes) 4 (no)
Number of children (mean)	2
Married	18 (4 widowed)
Community-dwelling	19

**Table 3:** Participants’ use of digital technology

Device	Yes	No
Smartphone	14	5
Computer	15	4
Emergency Button (RedCross)	1	18

**Table 4:** Participants’ reasons for NOT using smartphone technologies

Age group	Visually challenging (e.g. too small font/icons)	Discouraged by relatives	Cognitively challenging (e.g. too many functions)
90+		1	
80–89	1		2
70–79	1		1
60–69	-	-	-

### Interview data collection

 Participants were given the choice of conducting the interview at a venue of their choosing. All interviews took place either at the participant’s home or at the Felix-Platter Hospital. Before the start of the interview, participants were re-informed about the study and consent to participation.

The interview protocol (see Annex A) was developed by one author (MI) and validated by the entire research team. This semi-structured interview guide included a mix of semi-structured questions and informal prompts. The former question category, labeled “grand tour questions” by Leech (2002), was aimed at asking respondents to give a verbal tour of a certain topic [6]. The latter was only used in case the interviewees required further clarification related to the initial question or additional verbal cues to actively participate in the dialogue. Since interviews were carried out in different languages, the guide was followed but adapted based on linguistic specificities. Also, since most participants responded eloquently to our initial questions, prompts were used very rarely. This dynamic approach is consistent with best practices for semi-structured interviewing, which require researchers to ensure that the interview dialogue could “meander around the topics on the agenda –rather than adhering slavishly to verbatim questions as in standardized survey” [7].

All interviews started with questions about the interviewee’s daily life and his/her perceived sense of safety and wellbeing. Further, we explored the interviewee’s awareness about and previous experience with digital health technologies for healthy ageing. Finally, the interviewer addressed specific questions related to technology-mediated healthy ageing. This third portion of the interview protocol enveloped a series of demonstrations and subsequent user evaluation of the following technologies: a toy-shaped conversational robot (“Teddy” developed by SlowSoft AG), a smartphone application for care coordination (developed by Clever.Care AG), and two wrist-worn wearable devices (Apple Watch S4 developed by Apple Inc., and Fitbit Charge 3 developed by Fitbit Inc.). These technologies were selected as pertaining to three core domains of digital health for healthy ageing: socially assistive robotics, mobile health and wearable computing.

All interviews were conducted face-to-face by the same researcher [CS], a doctoral candidate at the time of the interviews, who had received trainings in qualitative data collection. Two more researchers [MI with background in bioethics and philosophy and TW with background in gerontology and bioethics] attended some of the interviews in order to minimize the risk of bias, to ensure quality-control and to assist the main interviewer in the detection of relevant non-verbal cues. There was no relationship between the participants and the researchers prior to the study. The interviews were recorded, and written notes were taken during the interviews. The interview took 50 min on average. No repeat interview was carried out. Transcriptions were offered to the participants and one participant requested his interview transcript for review.

### Data analysis

Audio-recorded data were transcribed verbatim with software assistance (*f4transkript* version 7 for Windows) and pseudonymized via code-assignation in order to ensure the privacy of the participants. Subsequently, transcripts were combined with the written notes taken during the interviews and entered into the MAXQDA software for computer assisted qualitative multimedia analysis (version 2018 for Windows). Written notes included observations related to the ease with which the participant reacted to the technology displayed to him/her. We ceased data collection after the 20th interview as it was clear to us that new interview data were not resulting in any novel findings compared to what we had already collected. Therefore, in agreeance with the criteria set by Fusch & Ness (2015) and Guest et al. (2006), we assessed that data saturation had been achieved [8, 9].

Step-by-step inductive thematic analysis [10, 11] was conducted, which led to several data-grounded themes. Transcripts were iteratively reviewed in light of new themes. To improve the validity and trustworthiness of the analysis, two researchers [MI and TW] took part in an auditing process to challenge the initial analyses through alternatives and counter examples until final themes and codes were agreed upon.

## Results

**Fig. 1:**
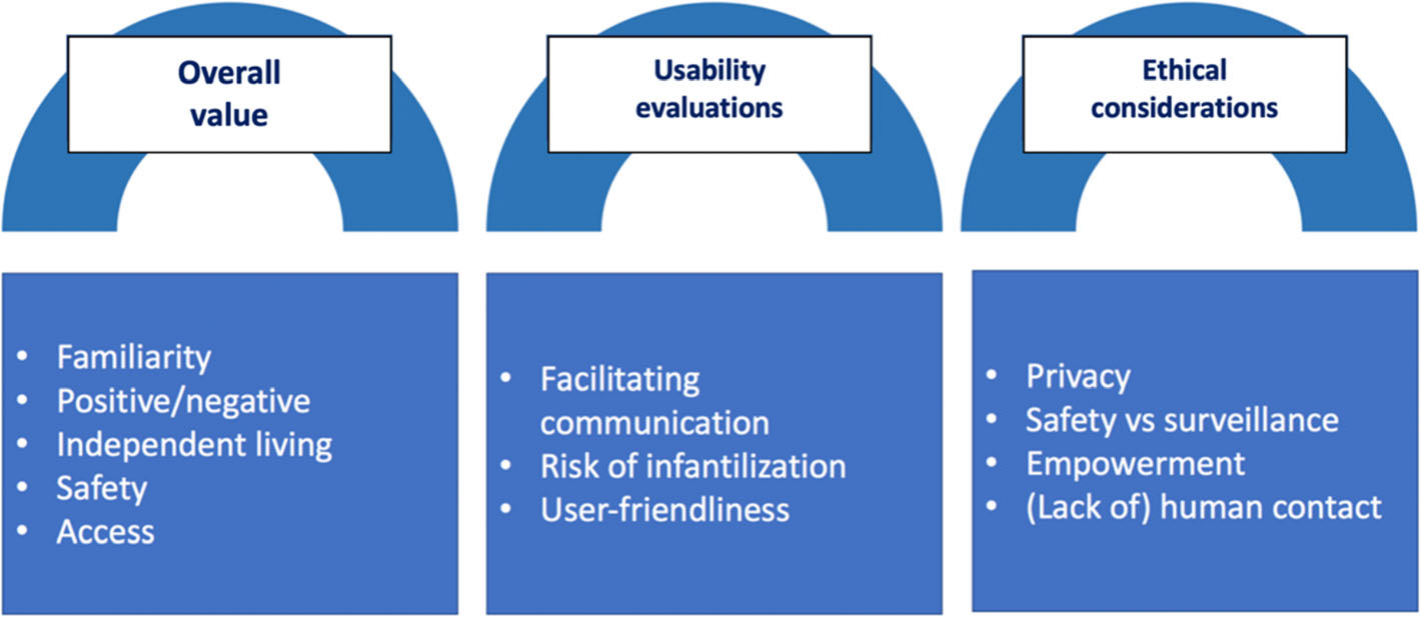
Overview of overall themes and subthemes

Inductive content analysis revealed three main themes and twelve sub-themes. The three main themes revolved around the following thematic areas: general value of digital assistive technologies, usability evaluations and ethical considerations. Figure 1 provides an overview of overall themes and subthemes.

The analysis showed that most participants were fairly familiar with existing digital health technologies for healthy ageing. Many of them reported to be frequent personal computer and smartphone users. However, even among our small study sample, we observed a generational gap in technology penetration and use between older adults aged between 65 and 79 and people aged 80 or older. In the latter subgroup, both device use and general digital awareness were lower or absent altogether. Most participants familiar with the use of smartphones and personal computers reported using these technologies already before retirement, especially in the workplace.

Most interviewees had a generally positive attitude towards digital health technologies and believed that digital tools could positively contribute to improving their overall wellbeing. No interviewee displayed an explicitly anti-technological stance. In particular, interviewees expected that technology use could improve their safety and empower them by promoting their autonomy. When further articulating the reasons of such technological optimism, interviewees shared hope that digital health technologies for healthy ageing could help them fulfil their wish to age in place and prolong their permanence at home [Participant 19: “I think being at home is the most important thing”].

Interviewees who considered themselves as physically and cognitively healthy perceived a less urgent need to use digital health technologies but argued that they would be willing to increase their technology use if this could help them ensure their safety [Participant 1: *“I don’t think it is necessary now. But if that was the case* (feeling *no longer safe*), *then I would try everything possible to avoid that. So I would try to be in my apartment as long as possible”*; Participant 8: *“And if you can keep your independence with such digital things for a while, I would think that’s great, yes, absolutely”*].

Safety emerged as a key concern, and a major motivational factor for digital technology use among the participating seniors. Interviewees feared that, as ageing progresses, they may become more vulnerable to everyday risks as a consequence of loneliness, memory lapses or simple distraction [Participant 10: *“If the radio or the TV is on and I forget to turn it off it’s no big drama. But if I forget to turn off the stove… well… so if some technology helps me live a year longer, either independently or on my own responsibility, then I would probably choose something like that, yes. As a help, yes*”]. However, while all interviewees highly valued safety, most of them appeared unwilling to increase their safety at any cost, but only compatibly with broader wellbeing considerations. For example, one interviewee criticized the safety-oriented paradigm of digital health technology and argued that such paradigm is rooted in a widespread unwillingness to accept our mortality and the vulnerability of the human condition [Participant 18: *“I believe that getting old is connected to becoming frail and lapsed. At some point you might fall for the last time and break your femur. And this is often the end. I believe that behind too much surveillance there is ultimately a non-acceptance of the mortality that is part of our lives”].*

Prompted by the interviewer, interviewees also discussed issues related to access to digital health technology and cost reimbursement. All interviewees argued that digital health technologies whose clinical effectiveness is scientifically proven should be reimbursed by compulsory health insurance according to the Swiss Federal Law on Health Insurance. Some interviewees observed that if the basic health insurance does not cover the cost of digital health technology for health ageing, then such technologies could amplify pre-existing socio-economic inequalities and result in poor technology adoption. It cannot be ruled out, however, that this latter theme may be interview-guide induced (see Limitations).

### Usability evaluations of digital health technologies

Usability assessment revealed generally positive attitudes towards the care coordination app and the wearable devices and a negative stance towards the conversational robot. Some of the participants found the toy-shaped robot cute or charming and appreciated the device’s ability to speak in Swiss-German dialect. However, most interviewees found that the stuffed toy aspect of the device (in the form of a teddy bear, hence the name *Teddy*) was infantilizing them [Participant 1: *“I find that a bit primitive and ridiculous! (…) Well, that seems to me a very primitive way to occupy myself. I hope I never need that. It looks cute but that’s all”*]. Some older participants even reported to be offended by the alleged “puerile” and “childish” aspect and voice of the robot. Indicative of this, is one interviewee’s decision to withdraw from the study due to the perceived infantilization induced by the device. The interviewee confirmed his desire to withdraw from the study even after the research team clarified that the study was aimed at collecting user feedback (including negative feedback) on the technologies, not to promote or market them. One participant argued that the conversational robot could raise the risk of deception, especially among older people with cognitive disabilities [Participant 18: *“Yes, this is deception, yes, it’s deception! Some deception factor is natural, natural, it’s is always inherent in all these things. That people take something for real because they can no longer assess correctly that it is actually not real at all. So that seems to me to be something very difficult”*]. Another interviewee had a more charitable perspective and argued that the Teddy may be helpful to people who are lonely and/or ‘have dementia’.

All interviewees had a favorable view on the care coordination app as they highlighted the importance of improving and facilitating communication between patients, family caregivers, physicians and ambulant formal caregivers. Interviewees observed that such care coordination could be particularly useful to facilitate the coordination of ambulant care through the so-called Spitex organizations (which some of them had already being confronted after a hospital stay and most of them having positive experiences), that are organizations in Switzerland providing home care to community-dwelling seniors by trained nursing and housekeeping personnel. From a usability perspective, they found the app interface intuitive and easy-to-use. They also observed that visual similarity with mainstream messaging platforms such as WhatsApp could facilitate ease of use, in virtue of their pre-existing familiarity with such platforms. At the same time, its similarity with conventional messaging platforms such as WhatsApp made interviewees question the “added-value” of an additional application. A few participants noted that they would not use the app because they either lacked technological competence or preferred communicating via phone call instead of messaging.

The two wrist wearable devices under assessment were all positively evaluated by the participants. There was, however, a strong preference for those wearables with larger screen size (and Apple Watch over the Fitbit). Further, visually impaired study participants reported difficulties with the interface and attributed them to the fact that those interfaces were probably designed for the younger generation, not with a senior population in mind. Managing messaging and other services from the smartwatch appeared difficult to most study participants. However, they positively valued simple safety-enhancing features such as the Emergency SOS function on Apple Watch. This feature allows users to make a call with local emergency services, automatically share the user’s GPS position and alert selected emergency contacts with a predefined text message. Most interviewees observed that such easy-to-use feature would increase their sense of safety when they are alone at home or on a hike.

### Ethical Considerations

The ethics assessment revealed four main themes: privacy, striking the right balance between safety and surveillance, empowerment and (lack of) human contact. With regard to privacy, even the most techno-friendly interviewers shared concern about preserving their private sphere from invasive uses of digital health technologies. Much of this concern regarded the risk that one technology used for some assistive purpose could be either repurposed or used to collect redundant information. They also shared concerns about the proportionality between the overall benefit of a certain assistive task and the volume and variety of data collected from them by the application [Participant 15: *“I mean, if I fall out of bed and that (app) sends a message, then that’s not something that greatly disturbs my privacy. But if it records everything, how you move, where you move, what you eat, what you drink, and whatever else can be recorded with these apps today… pulse, blood pressure, behavior etc. then it becomes much more problematic”*]. The risk of data misuse-especially the risk that personally identifiable digital data could be leaked, stolen or access by malicious third parties-was also a chief concern [Participant 19: “As soon that you simply give out your personal data, there is a big danger that this data will be misused”]. Some interviewee argued that, from their perspective, camera-based surveillance systems for home use (e.g. ambient assisted living technologies) raise greater risks of privacy invasion compared to wearable devices [Participant 8: *“I think in the bathroom, in the toilet, if you go there naked and anyway to the toilet and so on, I would not like to have that…. But if you fall then, well, then at least you have the watch, which you can still put into operation. But video equipment, especially in the bedroom and bathroom, no… I’ll pass on that… otherwise I would have no problems”*]. Many privacy concerns revolved around data and informational privacy. Interviewees showed a general willingness to share health-related data for health-related purposes, especially with health professionals [Participant 2: *“With the doctors I know that (my data) won’t be re-shared, that’s clear”;* Participant 8: *“When it comes to health and (the app) only documents that, I find it perfectly okay”*]. Interviewees also attributed great importance to the trust relationship between them and their healthcare professionals and/or medical institutions [Participant 6: *“To professor [name of the doctor] I would tell him everything, how things go etc. He probably knows most of it (laughs) and if he wants to know something, I tell him… An official institution can have my data. But not the general public”*]. Overall, interviewees rated privacy as a very important asset [Participant 14 : *“Privacy is, you could say, a sacred good to me*”]. One interviewee linked the privacy risks of digital health technologies with data acquisition by health insurers [Participant 15: *“Privacy I find something very important. I’ve always worked for it and I think that’s something essential for me. It’s that there’s not just a camera here… then somehow that goes to a health insurance company, to an insurer and they see what’s going on here. That’s why I also have trouble with these apps, because there I give so much price of my life and my attitude and about how I move, feed, how I react, that’s none of the insurers business”*].

When discussing issues of privacy, many interviewees reflected on the importance of striking the right balance between enhancing safety and minimizing privacy-violating surveillance. For example, one interviewee observed that although digital technologies could erode information privacy, they could also thereby increase their physical privacy by obviating the need for institutionalized care. This reflects a broad notion of privacy which envelops both physical and informational privacy. [Participant 18: *“I give up my privacy also when I am in a nursing home, don’t I? You can come and look at me at any time and come in and so on. (Sighs) I think if you become in need of care, become invalid, you automatically give up a part of your privacy”*].

Empowerment through independent living was another key ethical concern. Most interviewees stated frequently the wish to remain independent and age in place [Participant 6: *“Yes, especially the possibility to be at home longer and to be independent, I think that is something important, yes”*].

Many interviewees attributed great importance to the preservation of human contact in the old age, especially as part of the care relationship. They feared that the expanding use of digital health technologies could reduce human contact and eliminate care tasks which require human empathy and emotions [Participant 2: *“Yes, but then you don’t talk to the staff anymore, don’t you? I think communication with the caregivers is very important. I don’t think I would ever use the [robot]. I think I would just use it sometime. But you need people”*]. Several participants seemed to concur that not all care needs and ageing-related phenomena can be addressed using digital technologies. [Participant 10: “*You can not only talk about the digital world, (…) but about the practical, about the social, about the human and so on, where you can do something, where you can see each other, where you can play or walk or do something and not only digital”*].

## Discussion

Our findings suggest that older adults in our cohort are neither oblivious nor conceptually hostile to digital health technologies. They recognize the potential of these technologies to empower older adults, improve the quality of care, partly compensate for the decreasing proportion of care workers, and thereby promote healthy ageing. Our observation of a generational gap (between older-olds and oldest-old) in our participant group regarding technology penetration suggests that those participants that were confronted with digital technologies in their younger late fifties or early sixties were more likely to use those technologies in their old age. The interviewees’ generally positive attitude towards digital health technologies corroborates previous evidence on old people’s views on interactive and assistive technology [12–14]. Further, it confirms their often-reported wish to age in place [15]. From this perspective, effective digital health technologies for healthy ageing can be interpreted as an empowering factor and enabler of ageing in place.

The prominence of safety-related concerns indicates that older people see digital health technology not only as a facilitator of independent living but also as a risk-reducing tool. Even though cognitively and physically healthy interviewees saw less need for technology use at that point of their lives, all interviewees acknowledged that digital health technology could be a useful tool to achieve healthy ageing. Although such technologies were perceived as useful, safety issues associated with technology use were deemed important. Further, such safety considerations were never discussed in isolation. Interviewees contextualized safety in agreeance with broader wellbeing considerations. These considerations included promoting freedom, respecting autonomy, preserving privacy, improving health or even embracing human mortality and the vulnerability of the human condition. Value conflicts between safety and privacy were less prominent than generally expected based on previous literature on this topic [16].

Considerations related to access to digital health technology and cost reimbursement sparked heated debates, with all interviewees arguing that digital health technologies should be reimbursed by basic health insurance, provided that their clinical effectiveness is scientifically proven. These findings are in accordance with recent proposals to recognize access to available and affordable assistive technology as a basic human right to which all people with disabilities are entitled [17, 18]. This right has been argued both as a matter of compensatory justice and on the grounds of a non-discriminatory interpretation of the Convention on Rights of Person with Disabilities (CRPD). This is consistent with the interviewees’ concerns about the risk of discrimination and amplification of pre-existing socio-economic inequalities. The reason why interviewees wished reimbursement under compulsory private insurance lies in the fact that there are no free state-provided health services in Switzerland, but private health insurance is compulsory for all residents according to the Swiss Federal Law on Health Insurance.

Usability assessment revealed positive attitudes towards the care coordination app and the wearable devices and a negative stance towards the conversational robot. However, this finding is not generalizable to the entire field of conversational robotics. Previous research has shown that older people tend to have positive evaluations of conversational robots [19, 20]. The same body of research, however, has shown that participants’ positive attitudes towards conversational robots largely depended on facial appearance and are elicited prior to interacting with the robot. For this reason, we hypothesize that the appearance of the Teddy robot as a stuffed toy is the primary explanation for the interviewees’ generally negative perceptions. The strong resemblance to a toy has likely elicited feelings of infantilization. It should be highlighted that infantilization is a major ethical and psychological concern in the old age [21]. The risk of deception, in contrast, is not reducible to the toy-like semblance of the Teddy and raises broader ethical concerns which apply —albeit in different degrees— also to pet-shaped, machine-like and humanoid robots [22]. Further research should investigate if the toy shape can be more appreciated among older adults with dementia compared to cognitively healthy older adults.

Similarly, the generally positive views towards the care coordination could be partly motivated by the specifics of the Swiss healthcare system, in particular with the role of Spitex organizations in home care delivery. The term “Spitex” is an abbreviation for “external help and care” and is generally used in the Swiss-German language area to refer to ambulant care.

In Switzerland, Spitex organizations promote, support and enable with their services patients of all ages living at home. Through Spitex services, older people and people with medical needs can be supported and cared for to a certain degree at home in a familiar environment by trained nursing and housekeeping personnel. This is supposed to promote the autonomy and independence of the person. Spitex is often seen as a cost saving option compared to inpatient care in a nursing home, and the costs of the services are partly borne by the patient (or his/her health insurance company) and partly subsidized by the public sector (usually the municipality). For these reasons, it is possible that interviewees particularly favored a digital tool which could facilitate the delivery of such ambulant service.

The convergence of the interviewees’ ethics assessment revealed a strong consensus on ethical themes. With regard to privacy, our findings show a strong concern about preserving their private sphere from invasive uses of digital health technologies for healthy ageing. This concern also entails an appraisal of the proportionality between the privacy sacrifice caused by a certain digital intervention (e.g. in terms of the volume and variety of data collected from subjects) and the overall benefit for health and well-being of that intervention [23]. Participants appeared generally willing to sacrifice a portion of their privacy as trade-off to the benefit of safety and independent living, provided they could retain a sufficient degree of autonomy and self-determination regarding technology use. Nonetheless, several privacy concerns were raised, especially fears of data misuse which may have been fueled by recurrent data breaches and scandals [24, 25]. Such fears, however, did not appear to negatively affect the interviewees’ general willingness to share health-related data for health-related purposes, especially with trusted health professionals and medical institutions. Interviewees critically reflected on the importance of striking the right balance between enhancing safety and minimizing privacy-violating surveillance. In doing so, they often revealed a non-reductionist understanding of privacy which involves both physical and informational privacy. It remains an open question, however, whether and how these different forms of privacy can be compared and how it can be determined which form of privacy should be privileged (prioritized?).

Finally, the great importance attributed by interviewees to the preservation of human contact in the old age corroborates previous evidence from study with proxy stakeholders (formal and informal caregivers) on the implications of intelligent assistive technology for the care relationship [4, 5]. The risk that the expanding use of digital health technologies for healthy ageing could reduce human contact [26, 27] and eliminate care tasks which require human empathy and emotions was a frequent concern and, as such, requires careful attention.

These findings provide empirically grounded information for the design, development and implementation of digital health technologies for healthy ageing. First, they show that patient-centered technologies designed to promote freedom and independent living in the old age are likely to be adopted and appreciated by community-dwelling older adults. Furthermore, they show that ethical considerations are key determinants of acceptance and adoption. In particular, technologies capable of increasing safety without thereby disproportionately trifling (both physical and informational) privacy seem to emerge as the golden standard for assistive technologies in this domain. Finally, the general consensus about reimbursing the costs of digital assistive technology by basic health insurance solicits policy makers and insurers to consider cost-reimbursement and subsidy plans for clinically validated assistive technologies. These findings are in accordance with recognizing access to available and affordable assistive technology as a basic human right to which all people with disabilities are entitled as a matter of compensatory justice and on the grounds of a non-discriminatory interpretation of the CRPD.

## Limitations

Our qualitative study provides novel user-generated insights on the ethical assessment of digital health technologies for healthy ageing. Further, it provides a rich exploration of older people’s perspectives on such technologies which envelops both practical (e.g. usability-related) and speculative (e.g. ethical and experiential) considerations. However, the nature of the qualitative interview methodology presents several limitations. The first limitation pertains to sampling. In light of both the small sample size (*n* = 19) and purposive sampling strategy used in the study, our results are not statistically representative of the Swiss older population. In particular, given the relatively high degree of digital savviness of our interviewees, it is possible that our results may not be representative of the oldest-old segment of older people with poor digital literacy. Second, qualitative interviews carry a risk of social desirability and/or interviewer bias [28] as it cannot be excluded that the study participants prioritized the discussion of ethical issues that they believed the interviewer was interested in capturing. This implies that some other important considerations may have remained undiscussed. At the same time, the researcher solicited opinions on specific issues (e.g. reimbursement) hence the emphasis on certain topics could be in part an artifact of the question posed. For example, questions 26, 28 (especially the second part of the question) and 31 may have inadvertently induced or prompted a certain answer. It should be noted that the interview guide provided as supplementary document is a translation in English, while interviews were carried out in German or French, and that the guide was not followed statically during the interviews as the interviewer needed to phrase these questions based on the respondents’ responses and level of understanding. Finally, to minimize the risk of bias, however, we validated the interview protocol multiple times prior to data collection, and sought feedback from peer experts. Further, we minimized the risk of subjective bias by overseeing the early phase of data collection with two researchers, conducting several debriefing sessions and having at least two researchers analyze the data independently.

## Conclusions

Our study reveals a generally positive attitude towards digital health technologies as participants believed digital tools could positively contribute to improving their overall wellbeing, especially if designed in a patient-centered manner. Safety concerns and ethical issues related to privacy, empowerment and lack of human contact also emerged as key considerations. The results of our qualitative study highlight an informationally rich spectrum of end-user perspectives on digital technologies for healthy ageing. These findings complement previous ethics assessment studies involving proxy decision-makers such as informal and formal caregivers with first-hand information from older persons. This information can help the developers of technologies for digital ageing to identify relevant practical and ethical considerations that need to be incorporated into technology design. Further, it can contribute to identifying the requirements for user-centered ethical design in the domain of digital health technology for healthy ageing.

## Supplementary Information

**Additional file 1.**

## Data Availability

The datasets generated and/or analysed during the current study (audio files and written transcripts) are not publicly available due to privacy considerations but are available from the corresponding author on reasonable request.
