# Supplementary material for: Digital health interventions for healthy ageing: a qualitative user evaluation and ethical assessment
Source: BMC Geriatr. 2021 Jul 2;21:412. doi: 10.1186/s12877-021-02338-z (PMC8252216; doi:10.1186/s12877-021-02338-z)
Supplement: Supplementary file 1 — Additional file 1. [file 12877_2021_2338_MOESM1_ESM.doc]

# Digital Health Interventions for Healthy Aging: A Qualitative User Evaluation and Ethical Assessment

Authors: Marcello Ienca*1-2 , PhD; Christophe Schneble3 , M.Sc.; Reto W. Kressig4 , MD; Tenzin Wangmo3 , PhD

1 Department of Health Sciences and Technology, ETH Zurich

2 Competence Centre for Rehabilitation Engineering and Science, ETH Zurich

3 Institute for Biomedical Ethics, University of Basel

4 University Department of Geriatric Medicine FELIX PLATTER and University of Basel

* Corresponding author Correspondence to: Dr. Marcello Ienca, Department of Health Sciences and Technology, ETH Zurich, Hottingerstrasse 10, HOA H17.

Email: marcello.ienca@hest.ethz.ch

**Interview Guide**

**Project Title:** Digitalizing Elderly Care in Switzerland: Opportunites and Challenges

***The following open-ended questions will be posed to the study participants and based on their responses further probing questions will be asked.**

**Block 1: Einführung / Introduction**

**Question**

1. Wie geht es Ihnen heute? / How are you today?

(Falls Interview findet im Spital statt) Wie sind Sie heute zur Klinik gekommen?  / (If interview takes place at the hospital) How did you get to the clinic today?

(Falls Interview findet zu Hause statt) Wie läuft Ihr Tag so bisher?  / (If interview takes place at home) How's your day going so far?

**Question**

1. Können Sie uns einen typischen Tag von Ihnen beschreiben? Welche Unterhaltung nutzen Sie? (Z.B. Spiele, Fernsehen, usw.) / 2. Can you describe a typical day of yours? What kind of entertainment do you use? (e.g. games, television, etc.)

**Block 2: Betreuungserlebnisse und -bedürfnisse allgemein** / **Block 2: Care experiences and general needs**

**Question**

1. Welche Aktivitäten führen Sie im Alltag selber aus? / 3. Which activities do you carry out yourself in everyday life?

**Question**

1. Für welche Sachen brauchen Sie Hilfe? Erleben Sie Einschränkungen in Ihrem Alltag?  / 3. What things do you need help for? Do you experience limitations in your everyday life?

*Prompt: Erstmal auf Antwort warten. Falls die Interview-Person ein Prompt braucht:* *Mobilitätseinschränkungen? Gedächtnis? Physische Unterstützung?*/ Wait for an answer first. If the interviewee needs a prompt:

Mobility restrictions? Memory? Physical support?

**Question**

1. Bei welchen Handlungen brauchen Sie in Ihrem Alltag Unterstützung?  / 5. Which activities do you need support for in your everyday life?

*Prompt:* Zum Beispiel: Hilfe beim Essen? Auf Toiletten gehen? Mit den Betreuerinnen zu kommunizieren? Medikamenteneinnahme? Termine einhalten?

*Antwortoptionen: 1) Ja, regelmässig. 2) Ja, aber manschmal. 3) Nein, nie.*

*Prompt: For instance: Help with eating? Going to the toilets? To communicate with the caretakers? Taking medication? Keeping appointments?*

*Answer options: 1) Yes, regularly. 2) Yes, but sometimes. 3) No, never.*

**Question**

1. Und wer unterstützt Sie (z.B. Spitex, Angehörige) im Alltag? /
2. And who supports you (e.g. Spitex, relatives) in everyday life?

**Question**

1. Wie zufrieden sind Sie mit Ihrer Betreuung?

*Prompts:*

1 = Sehr zufrieden,  
2 = Zufrieden,  
3= Mittelmäßig,  
4= eher weniger
5= Gar nicht zufrieden

**Question**

1. How satisfied are you with your support?

Prompts:

1 = Very satisfied,

2 = Satisfied,

3= Mediocre,

4= rather unsatisfied

5= Not satisfied at all

**Question**

1. Was finden Sie gut? Was finden Sie schlecht? Was sollte man verbessern?  / 8. What do you like? What do you think is bad? What should be improved?

**Question**

1. Was würde Ihr Alltagsleben erleichtern/verbessern? (Bezug nehmen auf die Antworten von Frage 3) / What would facilitate/improve your everyday life? (Refer to the answers to question 3)
2. Wie sicher fühlen Sie sich, wenn Sie zu Hause sind?

*Prompts:*

1 = Sehr sicher,  
2 = Sicher,  
3= Mittelmäßig,  
4= weniger sicher,  
5= Gar nicht sicher

 Was würde Ihr Sicherheitsgefühl erhöhen?

**Question**

1. How safe do you feel when you're home?

Prompts:

1 = Very safe,

2 = Safe,

3= Mediocre,

4= relatively unsafe,

5= Not safe at all

What would increase your sense of safety?

**Block 3: Technische Hilfsmittel zu Hause & digitale Kenntnisse / Block 3: Technical aids at home & digital knowledge**

**Question**

1. Sind technische Hilfsmittel bei Ihnen im Einsatz? Wir meinen damit Dinge wie ein Smartphone, ein Tablet oder eine Notfall-Uhr. Wie ist Ihre Erfahrung damit?  / 11. Are you using technical aids? We mean things like a smartphone, a tablet or an emergency clock. What is your experience with them?

**Question**

1. (Nur für Smartphone-Users): Finden Sie es schwierig, Ihr Smartphone zu benutzen?

*Prompts:*

1 = Sehr schwierig,  
2 = Schwierig,  
3= Mittelmäßig,  
4= weniger schwierig,  
5= Gar nicht schwierig

Wenn ja, was finden Sie besonders schwierig? Die Tasten zu finden? Zu viele Funktionen?

**Question**

1. (For smartphone users only): Do you find it difficult to use your smartphone?

*Prompts:*

1 = Very difficult,

2 = Difficult,

3= Medium,

4= not so difficult,

5= Not difficult at all

If so, what do you find particularly difficult? Find the keys? Too many functions?

**Question**

1. Wissen Sie was Apps sind? Verwenden Sie einige? (Nur für Smartphone-Users) / Do you know what apps are? Do you use some? (For smartphone users only)

**Question**

1. Smartphone-User: Welche Apps benutzen Sie am häufigsten? Was ist Ihre Lieblings-App? Verwenden Sie jemals Ihr Smartphone für Gesundheitszwecke?  / 14 Smartphone users: Which apps do you use the most? What is your favorite app? Do you ever use your smartphone for health purposes?

**Question**

1. Wenn Sie einverstanden Sie, würden wir Ihnen gerne eine App zeigen. / If you agree, we would like to show you an app.

Technologie 1: 2min Demo + 2 min freie Interaktion (participant observation). / Technologie 1: 2min Demo + 2min freie Interaktion (Teilnehmerbeobachtung).

**Question**

1. Wie war Ihre Erfahrung damit? Finden Sie diese App interessant/ einfach zu nutzen? Was würden Sie ändern? / What was your experience with that? Do you find this app interesting / easy to use? What would you change?

**Question**

1. Haben Sie je einen Roboter gesehen? Wir zeigen Ihnen gerne einen (demo) / 17. Have you ever seen a robot? We would be happy to show you one (demo)

Technologie 2: 2min Demo + 2 min freie Interaktion (participant observation). / Technology 2: 2min Demo + 2 min free interaction (participant observation).

**Question**

1. Wie war Ihre Erfahrung damit? Finden Sie den Teddy interessant/ einfach zu nutzen? Was würden Sie ändern? / What was your experience with that? Do you find the Teddy interesting or easy to use? What would you change?

**Question**

1. Würden Sie sich gerne mit dem Teddy sprechen?

*Prompts:*

1 = Sehr gerne,  
2 = gerne,  
3= Mittelmäßig,  
4= ungerne,  
5= sehr ungerne

Was würde dafür oder dagegen sprechen?

19. Would you like to talk to the teddy bear?

*Prompts:*

1 = Very gladly,

2 = gladly,

3= medium,

4= rather not,

5= very reluctant

What would be the pros and cons?

**Question**

1. Worüber würden Sie gerne mit dem Teddy sprechen?  Auf welcher Sprache? Was würden Sie gerne von dem Teddy hören? /

What would you like to talk to the teddy bear about? In which language? What would you like to hear from the Teddy?

**Question**

1. Was denken Sie über diese Technologien, die wir Ihnen gezeigt haben? / What do you think about the technologies we have shown you?

**Block 4: Kommunikation** / **Block 4: Communication**

**Question**

1. Sie sagten, Sie werden im Alltag von X unterstützt (Frage 6). Wir würden gerne wissen, mit welchen Kommunikationsmitteln Sie mit Ihren Angehörigen / mit Ihren Spitex-Betreuerinnen kommunizieren.

*Prompts:*

1) Face-to-Face,

2) Telefon/Natel Anruf

3) SMS

4) Whatsapp

5)Skype

6) Andere?

**Question**

1. you said that you are supported by X in everyday life (question 6). We would like to know which means of communication you use to communicate with your relatives and Spitex caregivers.

*Prompts:*

1) Face-to-face,

2) Telephone/Natel call

3) SMS

4) Whatsapp

5)Skype

6) Others?

1. Wie effizient finden Sie diese Kommunikation?  How efficiently do you find this communication?

*Prompts:*

1= Sehr effizient,  
2 = effizient,  
3= Mittelmäßig,  
4= weniger effizient,  
5= Gar nicht effizient

1 = Very efficient,

2 = efficient,

3= Medium,

4= not so efficient,

5= Not efficient at all

**Question**

1. Würden Sie gerne öfter/besser mit ihnen kommunizieren?  / Would you like to communicate more often/better?

**Question**

1. Wir zeigen Ihnen gerne ein Produkt, um mit Ihren Angehörigen bzw. Betreuerinnen zu kommunizieren / We are happy to show you a product to communicate with your relatives or caregivers

Technologie 3: 2min Demo + 2 min freie Interaktion (participant observation). / Technology 3: 2min Demo + 2 min free interaction (participant observation).

**Block 5: Verbleib zu Hause und kognitive Assistenz / Block 5: Staying at home and cognitive assistance**

**Question**

1. Wie wichtig ist es für Sie, zu Hause zu bleiben anstatt ins Pflegeheim umzuziehen?

*Prompts:*

- 1. = Sehr wichtig,  
     2 = wichtig,  
     3= Mittelmäßig,  
     4= weniger wichtig,  
     5= Gar nicht wichtig

How important is it for you to stay at home instead of moving to a nursing home?

1 = Very important,

2 = important,

3= Medium,

4= not so important,

5= Not important at all

**Question**

1. Es gibt heute technische Hilfsmittel, welche Ihnen bei der Erinnerung unterstützen können. Vergessen Sie Ihre Medikamente zu nehmen oder den Herd auszuschalten, können Erinnerungen zu Ihnen geschickt werden und Unfälle wie z.B. Stürze verhindert werden. Der Teddy und viele Apps gehören dazu.

Was ist Ihr erstes Gefühl, wenn Sie von solch einer neuen Technologie hören?

*Prompts:* Neugierde? Angst? Skepsis? Gleichgültigkeit?  /

Today there are technical aids which can support you in remembering. If you forget to take your medication or switch off the stove, reminders can be sent to you and accidents such as falls can be prevented. The teddy bear and many apps are part of it.

What is your first feeling when you hear about such a new technology?

*Prompts:* Curiosity? Fear? Skepticism? Indifference?

**Question**

1. Wären Sie persönlich bereit, technischen Hilfsmittel wie der Teddy, Sensoren, Mikrophone, Kameras in Ihrer Wohnung zu akzeptieren, um Unfallrisiken zu minimieren?

*Prompts:*

1 = Sehr bereit,  
2 = bereit,  
3= Mittelmäßig,  
4= weniger bereit,  
5= Gar nichtbereit

Was würde dafür oder dagegen sprechen?

Would you personally be willing to accept technical aids such as the teddy bear, sensors, microphones, cameras in your home to minimize the risk of accidents?

*Prompts:*

1 = Very ready,

2 = ready,

3= Medium,

4= not so ready,

5= Not ready at all

What would speak for or against?

**Question**

1. Wären Sie bereit, Ihre Wohnung anders zu gestalten, z.B. mit technischen Hilfsmitteln, um länger und unabhängig zu Hause zu bleiben?  / 29. would you be willing to modify your home, e.g. with technical aids, in order to stay at home longer and independently?

**Question**

1. Ihrer Meinung nach, wer sollte für diese Produkte bezahlen?

*Prompts:*

1 = Sie
2 = Krankenkasse
3= Spitex
4= Andere

In your opinion, who should pay for these products?

1 = you

2 = Health insurance

3= Spitex

4= Other

**Question**

1. Würden Sie gerne einige Produkte, die wir Ihnen gezeigt haben, von Ihrer Krankenkasse bezahlt haben?

*Prompts:*

1 = Sehr gerne,  
2 = gerne,  
3= Mittelmäßig,  
4= lieber nicht,  
5= überhaupt nicht

Would you like to have some of the products we showed you paid for by your health insurance company?

1 = Very gladly,

2 = gladly,

3= Medium,

4= not really,

5= not at all

**Question**

1. Würden Sie technische Hilfsmittel als Eingriff in Ihre Privatsphäre einstufen? Würden Sie im Austausch für einen längeren Verbleib im eigenen Zuhause auf einen Teil ihrer Privatsphäre verzichten? / Would you classify technical aids as an invasion of your privacy? In exchange for a longer stay in your own home, would you forgo some of your privacy?

**Question**

1. Hätten Sie Empfehlungen, um digitale Lösungen für ältere Menschen zu verbessern? / Do you have any recommendations for improving digital solutions for older people?

**Question**

1. Haben Sie sonst noch Fragen? / 34. Any other questions?

Vielen Dank für Ihre Zeit. / Thank you very much for your time.
